# Supplementary material for: Diet Quality among Students Attending an Australian University Is Compromised by Food Insecurity and Less Frequent Intake of Home Cooked Meals. A Cross-Sectional Survey Using the Validated Healthy Eating Index for Australian Adults (HEIFA-2013)
Source: Nutrients. 2022 Oct 27;14(21):4522. doi: 10.3390/nu14214522 (PMC9655026; doi:10.3390/nu14214522)
Supplement: Supplementary file 1 [file nutrients-14-04522-s001.zip › nutrients-1944846-supplementary.pdf]

Table S1. Mean scores of Healthy Eating Index for Australian Adults (HEIFA-2013) by student characteristics from domestic and international students who completed two dietary recalls.[1]

**Student characteristics**	**Total (n = 93)**				**Domestic (n = 71)**				**International (n = 22)**			
	**N**	**Mean**	**95% CI**	**P**	**N**	**Mean**	**95% CI**	**P**	**N**	**Mean**	**95% CI**	**P**
Sociodemographic												
Age												
18-22 years	67	50.9	47.9-54.0	0.079	52	50.4	46.8-54.1	0.110	15	52.4	46.1-58.7	0.448
23-30 years	26	56.1	51.2-61.1		19	56.1	50.1-62.2		7	56.9	47.2-66.6	
Gender												
Female	75	51.9	48.9-54.8	0.434	58	51.5	48.0-55.0	0.577	17	53.1	47.4-58.8	0.592
Male	18	54.5	48.5-60.5		13	53.9	46.3-61.4		5	56.3	45.6-66.9	
Current academic degree												
Undergraduate	67	51.9	48.8-55.0	0.566	51	51.3	47.6-55.1	0.551	16	53.6	47.6-59.6	0.893
Postgraduate	26	53.6	48.6-58.6		20	53.5	47.5-59.5		6	54.4	44.3-64.5	
Marital status												
Never married	87	52.6	49.9-55.3	0.569	65	52.2	48.8-55.5	0.645	22	53.8	48.9-58.7	NA [2]
Married or other	6	49.5	39.1-59.9		6	49.5	38.6-60.5		0	NA	NA	
Current accommodation												
Parental home	54	51.4	48.0-54.9	0.400	52	50.8	47.1-54.5	0.234	2	69.4	53.7-85.1	NP [3]
Other than parental home	39	53.7	49.6-57.8		19	55.1	49.0-61.2		20	52.3	47.5-57.0	
Employment status												
No employment	31	54.6	50.0-59.1	0.242	18	55.1	48.7-61.4	0.259	13	54.0	47.5-60.6	0.912
Employed	62	51.3	48.1-54.5		53	50.9	47.2-54.5		9	53.5	45.6-61.4	
Weekly working hours												
Less than 20 hours	51	51.5	48.0-55.1	0.740	44	51.5	47.6-55.4	0.461	7	51.8	40.0-63.7	NP
20 hours or more	11	50.1	42.5-57.7		9	48.0	39.3-56.6		2	59.9	35.8-83.9	
Weekly income (AUD) [4]												
$500 or less	64	51.5	48.3-54.7	0.509	50	51.2	47.4-55.0	0.545	14	52.2	45.6-58.7	NP
More than $500	24	53.5	48.3-58.8		20	53.4	47.3-59.4		4	55.5	42.3-68.7	

Student characteristics	Total (*n* = 93)				Domestic (*n* = 71)				International (*n* = 22)			
	N	Mean	95% CI	P	N	Mean	95% CI	P	N	Mean	95% CI	P
Changes caused by COVID-19												
Living arrangement [5]												
No	80	51.4	48.6-54.2	0.070	64	51.2	47.9-54.5	0.170	16	52.2	46.5-58.0	0.281
Yes	13	58.4	51.4-65.3		7	58.6	48.5-68.6		6	58.1	48.7-67.5	
Job changes [6]												
No	61	52.1	48.9-55.4	0.789	45	51.9	47.9-55.9	0.988	16	52.7	46.8-58.5	0.442
Yes	32	52.9	48.4-57.4		26	52.0	46.7-57.2		6	56.9	47.3-66.4	
Difficulties in finding jobs												
No	65	52.8	49.6-55.9	0.652	51	52.5	48.7-56.2	0.602	14	53.9	47.4-60.3	0.979
Yes	28	51.5	46.7-56.3		20	50.6	44.6-56.6		8	53.7	45.1-62.4	
Employment income loss												
No	61	51.4	48.1-54.6	0.338	46	51.2	47.3-55.1	0.489	15	51.8	45.2-58.3	NP
Yes	23	54.4	49.1-59.6		20	53.6	47.8-59.5		3	59.5	44.6-74.3	
Received any government support payment												
No	57	53.4	50.0-56.7	0.409	39	53.7	49.4-58.0	0.264	18	52.6	47.2-58.1	NP
Yes	35	51.1	46.8-55.4		31	50.0	45.2-54.8		4	59.1	47.6-70.6	
Received any support payment from other sources												
No	88	51.9	49.2-54.6	NP	70	51.8	48.6-55.0	NP	18	52.1	46.8-57.4	NP
Yes	3	56.6	42.0-71.1		1	60.1	33.3-86.9		2	54.7	38.7-70.6	
Diet and health-related characteristics												
Food security status [7]												
Food secure	86	52.9	50.2-55.6	0.163	66	52.4	49.2-55.7	0.253	20	54.5	49.3-59.7	NP
Food insecure	7	45.9	36.4-55.4		5	45.3	33.4-57.2		2	47.4	30.9-63.8	
Does your accommodation provide meals? [8]												
No	32	55.0	50.6-59.4	NP	14	57.4	48.9-65.9	NP	18	52.8	47.6-58.1	NP
Yes	4	48.2	35.8-60.7		2	50.9	27.3-74.6		2	47.8	32.1-63.4	

Student characteristics	Total (*n* = 93)				Domestic (*n* = 71)				International (*n* = 22)			
	N	Mean	95% CI	P	N	Mean	95% CI	P	N	Mean	95% CI	P
Weekly food budget (AUD)												
$0-30	15	53.8	47.1-60.4	0.580	15	53.8	46.9-60.6	0.123	0	NA	NA	0.208
$31-60	26	52.2	47.1-57.2		21	50.2	44.5-55.9		5	60.9	50.6-71.1	
$61-100	39	53.5	49.4-57.6		28	54.8	49.8-59.7		11	50.0	43.2-56.8	
> $100	13	48.0	40.9-55.1		7	42.1	32.2-51.9		6	54.9	45.8-64.1	
Adequacy of cooking facilities												
Adequate	87	51.7	49.0-54.4	**0.046**	70	51.8	48.6-55.0	0.372	17	51.4	46.2-56.6	0.055
Inadequate	6	62.4	52.2-72.6		1	63.9	37.2-90.7		5	62.1	52.5-71.7	
Self-perceived cooking skills												
Excellent	16	57.1	50.8-63.4	0.180	12	57.6	50.0-65.2	0.119	4	55.8	43.6-68.0	0.908
Good	35	50.0	45.7-54.3		23	48.0	42.5-53.5		12	53.8	46.8-60.9	
Fair or poor	42	52.6	48.7-56.5		36	52.6	48.2-57.0		6	52.5	42.5-62.5	
Cooking frequency												
3 days/week or less	50	49.3	45.9-52.8	**0.013**	42	49.1	45.1-53.1	**0.033**	8	50.2	42.1-58.3	0.253
4+ days/week	43	55.9	52.2-59.7		29	56.1	51.2-60.9		14	55.9	49.8-62.0	
Eating out frequency												
3 days/week or less	85	52.7	49.9-55.4	0.486	66	52.1	48.7-55.4	0.804	19	54.8	49.5-60.1	NP
4+ days/week	8	49.3	40.3-58.4		5	50.5	38.1-62.8		3	47.4	34.0-60.9	
Self-rated physical health status												
Excellent or good	50	54.8	51.2-58.4	0.058	34	54.9	50.3-59.5	0.085	16	54.5	48.5-60.6	0.653
Average or poorer	43	49.6	45.7-53.5		37	49.2	44.9-53.6		6	51.9	41.8-62.0	
BMI category [9]												
Underweight	10	46.7	38.7-54.6	0.237	7	44.1	34.0-54.1	0.237	3	52.3	38.1-66.4	NP
Normal weight	65	53.7	50.5-56.8		48	53.2	49.4-57.1		17	54.8	49.0-60.7	
Overweight or obesity	18	50.9	44.8-57.0		16	51.5	44.7-58.4		2	47.7	30.7-64.6	
Self-rated mental health status												
Excellent or good	40	49.8	45.9-53.8	0.093	29	49.8	44.8-54.7	0.262	11	49.9	43.3-56.5	0.096
Average or poorer	53	54.3	50.9-57.8		42	53.4	49.3-57.5		11	57.7	51.1-64.4	

Student characteristics	Total (n = 93)				Domestic (n = 71)				International (n = 22)			
	N	Mean	95% CI	P	N	Mean	95% CI	P	N	Mean	95% CI	P
WHO-5 Well-being index category												
Normal	51	52.7	49.1-56.2	0.810	37	52.9	48.5-57.3	0.518	14	52.0	45.8-58.2	0.316
Poor wellbeing (below 13)	42	52.0	48.1-56.0		34	50.9	46.3-55.5		8	57.0	48.9-65.2	

[1] Mean scores adjusted for energy intake (EI):basal metabolic rate (BMR) through analysis of covariance (ANCOVA), the potential range of HEIFA-2013 was 0-100; [2] Not applicable; [3] Not enough power; [4] Weekly income, include employment and/or other income; [5] Living arrangement changes, e.g., moved to less expensive premises; [6] Job changes, e.g., lost employment or worked less hours; [7] Food security status was assessed by the 18-item Household Food Security Survey Module; [8] A question for students lived outside parental or own home; [9] BMI cut-offs, underweight (<18.5 kg/m^2), normal weight (18.5-24.9 kg/m^2), overweight (25.0-29.9 kg/m^2) and obesity (≥30.0 kg/m^2).
